# Acute kidney injury in pregnant women with decompensated liver cirrhosis, an uncommon but dangerous condition: a lesson for the clinical nephrologist

**DOI:** 10.1007/s40620-023-01834-2

**Published:** 2024-01-19

**Authors:** Rasha Samir Shemies, Tamer Zaki Gaber, Mohamed Mohamed Shawqi, Alaa Mosbah, Nagy Sayed-Ahmed, Giorgina Barbara Piccoli

**Affiliations:** 1 Mansoura Nephrology and Dialysis Unit, Mansoura University, Mansoura, Egypt; 2 Department of Emergency Medicine, Benha University, Benha, Egypt; 3 Department of Obstetrics and Gynaecology, Mansoura University, Mansoura, Egypt; 4 Nephologie Centre Hospitalier Le Mans, 72000 Le Mans, France

**Keywords:** Acute kidney injury, Pregnancy, Decompensated liver cirrhosis, Hepatitis C, Preterm delivery

## The cases

### Patient #1

A 30-year-old primigravida in septic shock, who had been diagnosed with autoimmune hepatitis and liver cirrhosis (Child–Pugh score 7) 2 years earlier, maintained on prednisone (15 mg/d) and azathioprine (100 mg/d), was referred to our tertiary care hospital two days after delivering a still-born baby (25 gestational weeks) following prelabour preterm rupture of the membranes (PPROM). A comprehensive history taken from her family revealed that she had tried to become pregnant for 6 years and once pregnant, had not sought regular antenatal care.

On physical examination, the patient was febrile (38.7 °C) and had jaundice and low blood pressure (88/50 mmHg). Her serum creatinine was 1.6 mg/dL; her urine output was 300 ml on the day of presentation, after which she became anuric. Laboratory data, ultrasounds and treatment are summarised in Table 1. The patient was admitted to the intensive care unit (ICU), where her condition rapidly deteriorated, and she died two days later.

**Table 1:** Summary of the main imaging, laboratory data and treatments

Patients	Patient #1	Patient #2	Patient #3
Ultrasounds	Normal kidneys, cirrhotic liver, moderate hepato-splenomegaly	Normal kidneys, cirrhotic liver, moderate splenomegaly, ascites	Normal kidneys, shrunken cirrhotic liver, moderate splenomegaly, ascites
Haemoglobin (g/dL)	8.5 (12.1–15.1)	10.5 (12.1–15.1)	10.1 (12.1–15.1)
Leucocyte count (10^3^/mm^3^)	44 (4–11)	4.5 (4–11)	7.3 (4–11)
Platelet count (10^3^/mm^3^)	102 (150–400)	70 (150–400)	94 (150–400)
Serum albumin (g/dl)	2.9 (3.5–5)	2.7 (3.5–5)	2.2 (3.5–5)
Serum bilirubin (mg/dl)	17 (0.2–1.2)	13 (0.2–1.2)	16.1 (0.2–1.2)
Prothrombin INR (PT-INR)	1.2 (0.8–1.1)	1.7(0.8–1.1)	1(0.8–1.1)
ALT (U/L)	91 (7–56)	230 (7–56)	40 (7–56)
AST (U/L)	122 (8–45)	330 (8–45)	32 (8–45)
S-creatinine at presentation (mg/dl)	1.6 (0.59–1.1)	1.5 (0.59–1.1)	1.5 (0.59–1.1)
S-creatinine at discharge (mg/dl)	2.3 (at death)	0.7	0.6
urinalysis	N/A	no abnormality detected	proteinuria + 2, RBCs 25/HPF, WBCs 20/HPF
Child–Pugh score	8	10	11
treatment	I.V. fluids, albumin 20% infusion (20g/d), vasopressors, antibiotics	I.V. albumin 20% (20g/d), furosemide (120 mg/d), antibiotics	methyldopa (750 mg /d), I.V. albumin 20% (20g/d), furosemide (160 mg/d)

*PT-INR* Prothrombin-International normalized ratio, *ALT* Alanine Aminotransferase, *AST* Aspartate Aminotransferase, *S-creatinine* serum creatinine, *RBCs* Red Blood Cells, *WBCs* White Blood Cells

### Patient #2

A 39-year-old woman, (gravida 8, para 3) with a history of four first-trimester spontaneous abortions, presented with PPROM at 34 gestational weeks. She vaginally delivered a baby weighing 2100 g, who was admitted to the neonatal ICU. At admission, the patient displayed jaundice and ascites; blood pressure was 110/70 mmHg, urinary output was 1200 ml/day and serum creatinine was 1.5 mg/dL. Chronic hepatitis C (HCV) infection and decompensated liver cirrhosis (Child–Pugh score 10) was first discovered during her eighth pregnancy, when she sought medical advice because of jaundice. Paracentesis excluded peritonitis. Further laboratory data, ultrasounds and treatment are reported in Table 1. After treatment with albumin infusion, diuretics, and paracentesis, the patient’s kidney function improved, reaching her baseline levels in 5 days, and both she and her baby were discharged in good general condition.

### Patient #3

After two days of oligo-anuria, a 38-year-old woman (gravida 8 para 7) ^−^presented to our institution at 33 gestational weeks. She had 7 children, all delivered at term. She had a history of liver cirrhosis secondary to HCV (Child Pugh score 9), diagnosed few months before pregnancy, however, the patient did not seek medical follow-up or attend regular antenatal care visits. On examination, the patient, who had been diagnosed with preeclampsia in view of hypertension (160/100 mmHg) and proteinuria (+ +), had tense ascites, jaundice, and anasarca; serum creatinine was 1.5 mg/dl (Table 1). At 34 gestational weeks, she vaginally delivered a baby weighing 2200 g, who was admitted to the hospital’s neonatal ICU. After supportive treatment with albumin infusion and diuretics (Table 1), the patient, with fully recovered kidney function and despite persistent signs of hepatic decompensation, and her baby were discharged 14 days later.

## Lessons for the clinical nephrologist

Pregnancy is rare in women with liver cirrhosis, as the disease generally occurs in older individuals, while in younger women the endocrine derangements associated with liver cirrhosis interfere with fertility. The physiological changes of pregnancy, i.e. expansion of the circulating blood volume and elevation of portal pressure, may strain an ailing liver. The few available literature reports describing maternal and foetal outcomes for women with cirrhosis, especially in the advanced stages of the disease, highlight an increased risk of complications, including hyperemesis gravidarum, placental abruption, preterm delivery, post- and intrapartum haemorrhage, and preeclampsia, however the maternal outcomes were variable among different studies [1–3].

The global burden of liver cirrhosis varies across the world, reflecting the differences in the distribution of underlying aetiologies and treatment strategies between high and medium–low income countries. The availability of antiviral agents has changed the epidemiology of HCV worldwide; however, the extent of the problem remains largely unknown in many parts of the world [4, 5]. The age-standardised death rate of cirrhosis was very high in Egypt in the period 1990 to 2017 [6]. Nonetheless, a decline in HCV-related cirrhosis is expected within the next 5–10 years following an ambitious screening and treatment programme enacted by the Egyptian government in 2014.

Pregnancy-related acute kidney injury (PR-AKI) has seldom been described in the context of liver cirrhosis, and may be underreported. To increase awareness on this rare cause of PR-AKI, we are reporting on this complication in three women with advanced liver cirrhosis caused by autoimmune hepatitis or hepatitis C infection. Patients 1 and 3 had normal kidney function before pregnancy, while the level of kidney function was not known for patient 2, who was first diagnosed with liver cirrhosis and kidney function impairment during pregnancy.

Liver cirrhosis is a major health threat leading to over one million deaths per year worldwide. Since severe cirrhosis is rare in young people, there are few reports regarding the complications it may cause in pregnancy [2, 3, 7]. A recent systematic review, including 2912 patients, reported maternal mortality in nearly 1% of pregnant women with cirrhosis (an 80-fold increase compared to pregnant controls without cirrhosis), mostly related to variceal haemorrhage during vaginal delivery in 70% of the cases [1]. Acute kidney injury was not included in the analysis of the complications and a search strategy combining “cirrhosis and pregnancy and (AKI or acute kidney injury or dialysis)” retrieved 87 results prior to August 16, 2023, none of which were relevant to the present discussion.

Since, with improving care, more women with liver cirrhosis are expected to seek to become pregnant, it is our hope that the series of pregnancy-related AKI we have reported will focus attention on this life-threatening occurrence.

In our cases, further decompensation of liver cirrhosis occurred in pregnancy, with jaundice, portal hypertension, oedema, and ascites. All presented with PPROM, also a risk factor of HCV transmission to newborns, and one presented with sepsis after intrauterine death.

Due to the overlap between AKI and preeclampsia, it is possible the former has been underreported, and in fact, one of our three patients was initially diagnosed as having preeclampsia.

Acute kidney injury is a well-known, life-threatening complication in patients with decompensated cirrhosis [2, 8]. Foreseeing its risk in pregnancy is important, since early identification of women at risk and access to antenatal care programmes are the only means we have to decrease the burden of complications.

In two patients, AKI was haemodynamic, and rapidly responded to albumin infusion, diuretics, and paracentesis. Sepsis may have played a role in the third case, in which the patient died. Sepsis is a leading cause of pregnancy-related AKI and mortality in developing countries [9], however, to date there are no reports on the combination of sepsis, cirrhosis, AKI and pregnancy.

Of note, none of the three women was counselled on the management and termination of pregnancy in view of their liver disease; neither patient with hepatitis-C related cirrhosis had received antiviral treatment before pregnancy. Patient 2 was first diagnosed during pregnancy and patient 3, who was overwhelmed by her 7 children, was non-compliant to seek medical treatment. Furthermore, none had undergone regular prenatal care visits. In the study setting, women with liver disease are often reluctant to seek medical care, and this might have contributed to late referral and PR-AKI in our three cases.

As a lesson for the clinical nephrologist  we may retain that pregnancy in women with liver cirrhosis is rare; decompensation is a threat in pregnancy, and AKI, as in the cases reported here, may follow. Late referral, and the lack of prenatal care underline the need for dedicated programmes for high-risk pregnancies.

## Data Availability

All our data are included in this study.

## Declarations

### Duplicate publication

The content of the manuscript has not been published or submitted for publication elsewhere.
